# Influence of Foliar Zinc Application on Cadmium and Zinc Bioaccessibility in *Brassica chinensis* L.: In Vitro Digestion and Chemical Sequential Extraction

**DOI:** 10.3390/foods13152430

**Published:** 2024-08-01

**Authors:** Lin Wang, Xueying Tao, Chang Liu, Xuefeng Liang, Yingming Xu, Yuebing Sun

**Affiliations:** 1 Innovation Team of Heavy Metal Ecotoxicity and Pollution Remediation, Agro-Environmental Protection Institute, Ministry of Agriculture and Rural Affairs, Tianjin 300191, China; wanglin2017@caas.cn (L.W.); taoxueying1029@163.com (X.T.); liuch596@mail2.sysu.edu.cn (C.L.); liangxuefeng@caas.cn (X.L.); ymxu1999@126.com (Y.X.); 2 Key Laboratory of Original Agro–Environmental Pollution Prevention and Control, Ministry of Agriculture and Rural Affairs, Tianjin 300191, China

**Keywords:** cadmium, zinc, foliar fertilizer, pakchoi, bioaccessibility, chemical form

## Abstract

Foliar zinc (Zn) application can affect the accumulation and bioaccessibility of cadmium (Cd) and Zn in crops. However, the mechanisms by which foliar Zn application influences Cd and Zn bioaccessibility remain elusive. This study examined the effects of spraying ZnSO_4_ and ZnNa_2_EDTA on bioaccessibility and chemical forms of Cd and Zn in pakchoi (*Brassica chinensis* L.) shoots and evaluated human health risks via pakchoi consumption. Spraying ZnSO_4_ reduced the concentrations of ethanol-extractable (F_ethanol_) and deionized water-extractable (F_d-H2O_) Cd, as well as the corresponding bioaccessible Cd concentrations (20.3–66.4%) and attendant health risks of Cd, whereas spraying high-dose ZnNa_2_EDTA significantly increased the concentrations of both Cd forms and bioaccessible Cd. Spraying ZnSO_4_ and high-dose ZnNa_2_EDTA significantly increased the concentrations of Zn in F_ethanol_ and F_d-H2O_ and the corresponding bioaccessible Zn concentrations (0.8–8.3-fold). F_ethanol_ and F_d-H2O_ were the primary sources of bioaccessible Cd and Zn, contributing more than 59% of the bioaccessible Cd and Zn. These results indicate that foliar Zn application can affect Cd and Zn bioaccessibility in pakchoi mainly by modulating Cd and Zn in F_ethanol_ and F_d-H2O_. These findings provide scientific support for the development of more efficient measures to produce safe and high-quality leafy vegetables from Cd-polluted soils.

## 1. Introduction

As a highly toxic heavy metal, cadmium (Cd) poses a major health threat to humans via the food chain, which was classified as a Group 1 carcinogen [1]. In addition, excessive exposure to Cd can cause multiple health problems including renal damage, hepatotoxicity, osteoporosis, and emphysema [2]. For nonsmokers, diet is the main route of Cd exposure [3]. In most countries, contaminated crops, particularly vegetables and cereals, account for most dietary Cd intake [2,3,4]. Oskarsson et al. [5] reported that vegetable consumption contributed to 83% of total Cd intake for the people residing on farms in southern Sweden. Therefore, reducing dietary Cd intake from vegetables is important to ensure food safety and protect human health.

Lowering total Cd concentrations in vegetables is the most direct strategy to reduce Cd exposure via vegetable consumption. Many approaches have been tested to reduce Cd accumulation in vegetables, among which foliar zinc (Zn) application has proven to be a rapid and economical method [2,6]. Owing to the similar characteristics of Cd and Zn, Zn can decrease Cd accumulation in plants by inhibiting Cd uptake and transport [7]. In addition, foliar Zn application can effectively increase Zn concentrations in vegetables, and thus ameliorate human Zn deficiency [8]. Therefore, in Cd-polluted farmlands, foliar Zn application can produce dual health benefits for humans.

Dietary Cd intake from vegetables is determined not only by Cd concentrations in vegetables, but also by Cd bioaccessibility. Bioaccessibility refers to the proportion of metal released from foods in the gastrointestinal environment and available for further absorption [9,10]. To accurately assess the health risks via dietary exposure to Cd, Cd bioaccessibility in foods should be considered. Recent studies have highlighted the influence of foliar zinc application on metal bioaccessibility in crops. For instance, Tang et al. [11] reported that foliar spray with a synthesized Zn fertilizer reduced the bioaccessible Cd concentration in a low-Cd-accumulating cultivar of water spinach (*Ipomoea aquatica* Forsk.) by 19.7% and increased the Zn bioaccessibility in a high-Cd-accumulating cultivar by 10.6%. Tao et al. [12] observed that foliar spray with Zn sulfate (ZnSO_4_) at a concentration of 12 mM significantly reduced Cd bioaccessibility in wheat (*Triticum aestivum* L.) grains in the gastric phase by 14.1%, and significantly increased Zn bioaccessibility in the gastric phase by 12.2%. Lin et al. [13] found that spraying ZnSO_4_ (0.05% Zn) significantly decreased the bioaccessible Cd concentration in rice *(Oryza sativa* L.) grains and had an insignificant effect on the bioaccessible Zn concentration. However, up to now, we still know little about the health risks via ingestion of Cd-contaminated vegetables treated with foliar Zn application, and the mechanisms underlying the influence of foliar Zn application on Cd and Zn bioaccessibility in vegetables remain elusive.

Recent studies have indicated that the speciation of arsenic (As) and mercury (Hg) in rice affects their respective bioaccessibility, and inorganic species of As and Hg are more bioaccessible than their organic species [14,15,16]. In addition, Liao et al. [14] also found that cooking could increase the proportions of organic As and Hg species in rice, thus reducing their bioaccessibility. Recently, a sequential extraction procedure has been developed, which can fractionate Cd and Zn in plant tissues into five operationally defined fractions [17,18]. Chemical forms of Cd and Zn can affect their mobility in plants, among which the soluble metals in inorganic form and organic form migrate more readily than the insoluble forms of metals, including pectate-/protein-integrated metals, insoluble metal phosphate, and metal oxalate [19]. However, few studies so far have examined the relationships between chemical forms of Cd and Zn and their bioaccessibility in plants. Clarifying the link between these factors may improve our understanding of how Cd and Zn bioaccessibility changes under different treatments.

As an important leafy vegetable, pakchoi (*Brassica chinensis* L.) has been cultivated and consumed worldwide. However, pakchoi tends to accumulate more Cd than other vegetables [20]. Given the high toxicity of Cd and its classification as a Group 1 carcinogen [1], reducing its bioaccessibility in pakchoi shoots is crucial for public health. While several studies have examined the effect of Zn application on Cd accumulation and bioaccessibility in plants, few have investigated the specific changes in the bioaccessibility and chemical forms of these metals in pakchoi. In this study, two cultivars of pakchoi with different Cd accumulation characteristics were planted in a Cd-contaminated soil and fertilized with ZnSO_4_ and Zn disodium ethylenediaminetetraacetic acid (ZnNa_2_EDTA) by foliar application. This study aims to (1) assess the impact of foliar Zn application on the bioaccessibility of Cd and Zn in pakchoi shoots and evaluate the bioaccessibility-based health risks of Cd associated with pakchoi consumption; (2) investigate the chemical forms of Cd and Zn under different treatments; and (3) elucidate the relationships between the bioaccessibility and chemical forms of Cd and Zn.

## 2. Materials and Methods

### 2.1. Plant Materials, Zn Fertilizers, and Soil

Based on our previous research [20], a low-Cd-accumulating cultivar (Huajun 2) and a high-Cd-accumulating cultivar (Hanlv) of pakchoi were grown in this work. ZnSO_4_ and ZnNa_2_EDTA for foliar application were of analytical grade (Shanghai Macklin Biochemical Co., Shanghai, China).

The tested soil was collected from the plow layer (0–20 cm) of a vegetable plot located nearby a Pb and Zn smelter in Jiyuan City, Henan Province, China. After collection, the soil was dried and ground to <5 mm. The soil was classified as Fluventic Ustochrept. The physicochemical characteristics of the soil were measured according to the analytical methods described by Bao [21] and are listed in Table S1.

### 2.2. Pot Experiment

A pot experiment was performed in a glasshouse in Tianjin (39°5′53″ N, 117°9′8″ E) from May to July 2020. The greenhouse was maintained at a temperature range of 20–35 °C with 50% humidity and natural sunlight. Each plastic pot (21 cm in diameter and 15 cm in height) contained 2 kg of soil, and urea (150 mg/kg nitrogen) and dipotassium hydrogen phosphate (45 mg/kg phosphorus and 114 mg/kg potassium) were applied as basal fertilizers. Approximately 30 pakchoi seeds were sown in each pot. Within 15–20 days (d) after sowing, seedlings were gradually thinned to five individuals in each pot. During the growth period, irrigation was conducted daily using deionized water to maintain normal growth.

The foliar Zn treatments were: (1) CK, deionized water; (2) S1, 4 mM ZnSO_4_; (3) S2, 12 mM ZnSO_4_; (4) E1, 1.33 mM ZnNa_2_EDTA; and (5) E2, 4 mM ZnNa_2_EDTA. Because the preliminary experimental results showed that foliar application of more than 5 mM ZnNa_2_EDTA could significantly inhibit the growth of pakchoi, we had to reduce the doses of applied ZnNa_2_EDTA in this study. The treatments were executed using a completely randomized block design with three replications. At the 49th, 55th, and 61st d after sowing, the foliar Zn treatments were applied thrice using a calibrated sprayer at a rate of 9 mL per pot after sunset, ensuring even coverage of the leaves. The measure was taken to prevent soil contamination by covering the soil surface during spraying.

At the 70th d after sowing, whole plants were harvested and separated into shoots and roots. Shoot samples were washed thoroughly with deionized water and superficially dried with filter paper. Fresh shoot samples were weighed and stored at 4 °C. The shoot samples were cut into small pieces with a ceramic knife, mixed well, and divided into four parts, which were used for the determination of total concentrations, chemical forms, and bioaccessibility of Cd and Zn, as well as for the combined test of in vitro digestion and sequential extraction.

### 2.3. Measurement of Total Concentrations of Cd and Zn

Fresh shoot samples (4.0 g) were oven-dried at 75 °C for 48 h, and then digested with 9 mL HNO_3_ in an ED54 digestion system (LabTech Inc., Beijing, China) [12]. Concentrations of Cd and Zn in the digested solutions of shoot samples were measured by inductively coupled plasma mass spectrometry (ICP-MS, iCAP Qc, Thermo Fisher Scientific, Waltham, MA, USA). During analysis of plant samples, reagent blanks and standard reference material (spinach leaves, SRM 1570a, National Institute of Standards and Technology, Gaithersburg, MD, USA) were included for quality control. The recovery rates of Cd and Zn in standard reference samples were 95.6–104.8% and 97.5–105.9%, respectively.

### 2.4. Extraction of Chemical Forms of Cd and Zn

Cd and Zn in pakchoi shoots were fractionated using the sequential extraction method described by Xin et al. [22] with slight modifications. The procedure separated Cd and Zn into five chemical forms by using the specific solutions in the following order: inorganic metals extracted with 80% ethanol (F_ethanol_), water-soluble metal complexes of organic acid and M(H_2_PO_4_)_2_ extracted with deionized water (F_d-H2O_), protein- and pectate-integrated metals extracted with 1 M NaCl (F_NaCl_), undissolved metal phosphate extracted with 2% CH_3_COOH (F_HAc_), and metal oxalate extracted with 0.6 M HCl (F_HCl_) [17].

The fresh shoot sample of pakchoi (5.0 g) was ground with a mortar and pestle, and extracted twice with 25 mL of extraction solution at 25 °C for 12 h each. After centrifugation at 12,000× *g* for 5 min, the two supernatants were collected and pooled. The retained plant material was extracted with the next solution using the same extraction procedure. All the pooled supernatants were heated to dryness at 75 °C, digested, and analyzed for Cd and Zn concentrations as described in Section 2.3.

To check the accuracy of the extraction results, the percent recovery of metal was calculated based on the ratio between the sum of metal concentrations in the five chemical forms and the total metal concentration. The percent recovery of Cd and Zn ranged from 96.4% to 99.7% and 95.1% to 100.0%, respectively, which indicates that loss of Cd and Zn during the sequential extraction was low.

### 2.5. In Vitro Gastrointestinal Digestion

The bioaccessible concentrations and bioaccessibility of Cd and Zn were measured according to the physiologically based extraction test (PBET) with slight modifications [9,23]. The PBET assay included the gastric and small intestinal phases. During the gastric phase, the fresh shoot sample (3.0 g) was ground and then extracted with 30 mL of gastric solution (0.50 g/L citrate, 0.50 g/L malate, 0.42 mL/L lactic acid, 0.50 mL/L acetic acid, and 1.25 g/L pepsin, pH 1.5) at 37 °C for 1 h. After centrifugation at 5000× *g* for 10 min, 3 mL of the supernatant was collected, filtered (0.45 μm), and stored at 4 °C as the gastric juice. During the small intestinal phase, 15 mg pancreatin and 1 mL of bile salt solution (52.5 g/L) were added to the rest of the mixture after increasing the pH to 7.0 using NaHCO_3_ powder. After incubation at 37 °C for 4 h and then centrifugation, a 3 mL aliquot was collected as the small intestinal juice. The Cd and Zn concentrations in the gastric and small intestinal juices were determined by ICP-MS.

To verify the accuracy of the digestion results, ten shoot samples (selected from five foliar treatments and two pakchoi cultivars) were extracted using the aforementioned digestion method, and the resultant sample residues after the small intestinal digestion were oven-dried, digested, and analyzed for Cd and Zn concentrations as described in Section 2.3. The recovery rate of metal was calculated by dividing the sum of amounts of metal in the gastric juice, small intestinal juice, and sample residue by the total amount of metal in the shoot sample. The recovery rates of Cd and Zn in the ten shoot samples were 94.4–102.7% and 95.6–101.5%, respectively, which shows that loss of Cd and Zn during in vitro digestion was small.

### 2.6. The Combined Test of In Vitro Digestion and Sequential Extraction

To confirm the possible contributions of Cd and Zn in F_ethanol_, F_d-H2O_, and F_NaCl_ to bioaccessible Cd and Zn in pakchoi shoots, shoot samples under treatments CK, S2, and E2 were selected for the combined test. To save time, we did not use all treatments for the combined test. The fresh shoot sample (15.0 g) was homogenized using a mortar and pestle. Two parts of the shoot sample (5.0 g each) were extracted by two steps (80% ethanol and deionized water) and three steps (80% ethanol, deionized water, and 1 M NaCl), respectively. The resulting residue after three-step extraction was washed with deionized water twice to remove the residual NaCl. The collected residues and another part of the shoot sample (3.0 g) were freeze-dried at −40 °C for 48 h. Then, the three samples were extracted with the gastric and small intestinal solutions to measure the bioaccessible concentrations of Cd and Zn.

### 2.7. Data and Statistical Analyses

The bioaccessibility of Cd and Zn was calculated as follows:

Bioaccessibility (%) = BAC/TC × 100%(1)

where BAC refers to the bioaccessible metal concentration and TC refers to the total concentration of metal in pakchoi shoots.

To evaluate the health risks of Cd via pakchoi consumption, the estimated daily intake (EDI) was calculated as follows:

EDI = (C × IR)/BW(2)

where C refers to the total Cd concentration or bioaccessible Cd concentration in the small intestinal phase in pakchoi shoots (μg/g). IR is the average daily consumption of pakchoi (g/day). BW refers to the average body weight (bw) of adults (kg). According to two surveys conducted in Shanghai and Jiangsu province, the IR and BW were 70.0 g/day and 63.2 kg, respectively [24,25].

The contribution rates (CR) of Cd and Zn in F_ethanol_, F_d-H2O_, and F_NaCl_ to bioaccessible Cd and Zn in pakchoi were calculated as follows:

CR_e+d_ (%) = (BAC_shoot_ − BAC_shoot-Fe+d_)/BAC_shoot_ × 100% (3)

CR_NaCl_ (%) = (BAC_shoot-Fe+d_ − BAC_shoot-Fe+d+N_)/BAC_shoot_ × 100% (4)

where BAC_shoot_ refers to the bioaccessible concentrations of Cd or Zn in shoot samples, and BAC_shoot-Fe+d_ and BAC_shoot-Fe+d+N_ refer to the bioaccessible concentrations of Cd or Zn in shoot sample residues after removing F_ethanol_ and F_d-H2O_, as well as F_ethanol_, F_d-H2O_, and F_NaCl_ by using sequential extraction, respectively.

Data were analyzed using SPSS Statistics 22 (IBM, Armonk, NY, USA) and Canoca 5 (Microcomputer Power, New York, NY, USA). The differences among foliar application treatments were assessed using one-way analysis of variance followed by Duncan’s multiple-range test at *p* < 0.05. Pearson correlation coefficients, stepwise multiple linear regression analysis, and redundancy analysis (RDA) were used to examine the relationships between chemical forms and bioaccessibility of Cd and Zn. All graphs were created using Origin 2022 (OriginLab Co., Northampton, MA, USA).

## 3. Results and Discussion

### 3.1. Cd and Zn Concentrations in Pakchoi Shoots

**Figure 1:**
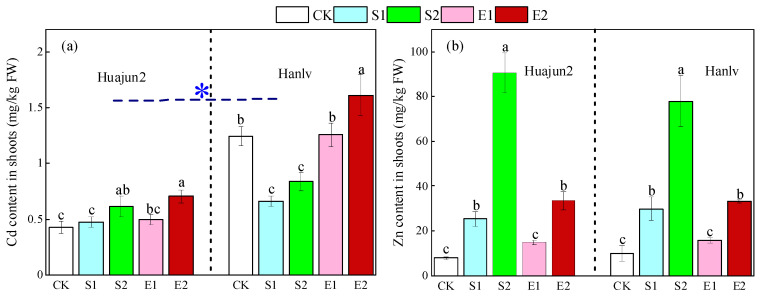
Concentrations of Cd (**a**) and Zn (**b**) in the shoots of the two cultivars of pakchoi. Different lowercase letters within the same cultivar denote significant differences amongst treatments at *p* < 0.05. The asterisk denotes Cd concentrations in the shoots of Huajun 2 differed significantly from those of Hanlv based on the paired-sample *t*-test (*p* < 0.05). CK, S1, S2, E1, and E2 represent foliar treatments applied with deionized water, 4 mM ZnSO_4_, 12 mM ZnSO_4_, 1.33 mM ZnNa_2_EDTA, and 4 mM ZnNa_2_EDTA, respectively.

Figure 1 shows the concentrations of Cd and Zn in pakchoi shoots under different treatments, highlighting significant differences visually. Foliar spray with the two Zn salts had different effects on Cd concentrations in the shoots of the two cultivars of pakchoi. The foliar application of low- and high-dose ZnSO_4_ significantly reduced Cd concentrations in the shoots of Hanlv by 46.7% and 32.9% compared to the control, respectively, whereas the foliar application of high-dose ZnSO_4_ significantly raised the Cd concentration in the shoots of Huajun 2 by 43.5%. Foliar spray with high-dose ZnNa_2_EDTA significantly raised Cd concentrations in the shoots of Huajun 2 and Hanlv by 29.5% and 63.9%, respectively. These results indicate that the influence of foliar Zn application on Cd accumulation in pakchoi depends on the form and dose of the applied Zn, as well as on the cultivars tested. Similarly, a previous study showed that the application of high-dose Zn reduced antioxidant enzyme activity and caused membrane damage, which resulted in increased Cd translocation in wheat [26]. Furthermore, Zn application can affect Cd translocation and accumulation in plants by regulating the expression of genes related to Cd transportation; the responses of the related genes are genotype-specific, which promote or inhibit Cd accumulation in different cultivars [7]. In addition, Cd concentrations in the shoots of Huajun 2 were significantly lower than those of Hanlv, which is attributed to the low Cd accumulation characteristic of Huajun 2 [27].

Foliar Zn application markedly enhanced Zn accumulation in pakchoi shoots compared to the control (Figure 1b). Specifically, the application of ZnSO_4_ significantly increased shoot Zn concentrations in the tested cultivars by 2.0–10.5-fold. The addition of high-dose ZnNa_2_EDTA also significantly increased Zn concentrations in the shoots of Huajun 2 and Hanlv by 3.2- and 2.4-fold, respectively. Interestingly, when the same level of Zn was applied, there were no significant differences in shoot Zn concentrations between treatments S1 and E2 in the two cultivars. This result is consistent with the finding of Haslett et al. [28], but differs from that of Wei et al. [29], who observed that the Zn concentration in rice grains under ZnSO_4_ treatment was significantly higher than that under ZnNa_2_EDTA treatment. The differences in these results may arise from the varied doses of Zn applied and the diverse organs of the plants measured.

### 3.2. Chemical Forms of Cd and Zn in Pakchoi Shoots

Different chemical forms of Cd in pakchoi shoots are presented in Table 1. Compared with CK, ZnNa_2_EDTA application significantly raised the concentrations of Cd in F_ethanol_ in Hanlv by 0.5- and 1.9-fold for the low and high doses, respectively. The concentrations of Cd in F_d-H2O_ significantly decreased with ZnSO_4_ application for Hanlv by 63.5% and 71.1% for the low and high doses, respectively, but significantly increased with ZnNa_2_EDTA application for Huajun 2 and Hanlv by 2.1–3.5-fold and 0.9–2.0-fold, respectively. ZnSO_4_ application significantly raised the concentration of Cd in F_NaCl_ in Huajun 2 by 73.3% for the high dose, while significantly reducing the concentrations of Cd in F_NaCl_ in Hanlv by 19.6% and 42.0% for the low and high doses, respectively. In contrast, ZnNa_2_EDTA application caused significant decreases in the concentrations of Cd in F_NaCl_ in Huajun 2 and Hanlv (54.4–56.7% and 54.3–68.0%, respectively). Zn application had an insignificant effect on the concentrations of Cd in F_HAc_ and F_HCl_ in the two cultivars.

Under the CK treatment, Cd was mostly in F_d-H2O_ and F_NaCl_, for these two chemical forms accounted for 83.1–90.8% of the total Cd in the shoots (Figure S1a). Consistent with the finding of Xue et al. [30], this result indicates that most of the Cd in the shoots of pakchoi is integrated with organic acids, proteins, and pectates containing abundant carboxyl and thiol groups that can tightly bind Cd [27,31]. ZnSO_4_ application substantially raised the proportion of Cd in F_NaCl_ and reduced that in F_d-H2O_, whereas ZnNa_2_EDTA application markedly raised the proportion of Cd in F_d-H2O_ and reduced that in F_NaCl_.

The concentrations of Zn in different chemical forms were evaluated in the shoots of the tested pakchoi cultivars (Table 2). Foliar Zn application significantly raised the concentrations of Zn in F_ethanol_ and F_d-H2O_ by 0.8–5.5- and 1.0–6.4-fold compared to the control treatment, respectively, except for the Zn in F_d-H2O_ in Hanlv under treatment E1. The concentrations of Zn in F_NaCl_ significantly increased with the foliar application of high-dose ZnSO_4_ for Huajun 2 and Hanlv by 15.9- and 10.5-fold, respectively. The foliar application of ZnSO_4_ significantly raised the concentrations of Zn in F_HAc_ and F_HCl_ by 3.7–18.0- and 2.2–6.9-fold, respectively. The concentrations of Zn in F_HAc_ and F_HCl_ in Huajun 2 significantly increased with the foliar application of high-dose ZnNa_2_EDTA by 3.9- and 3.1-fold, respectively.

Under the control treatment, Zn accumulated mainly in F_ethanol_ and F_NaCl_, followed by F_d-H2O_ and F_HAc_ (Figure S1b). This result is in line with previous works on Zn speciation in leafy vegetables using the synchrotron-based X-ray absorption near-edge structure (XANES), which showed that the majority of Zn was integrated with organic acids, amino acids, and phosphates [32,33]. The application of ZnSO_4_ reduced the proportions of Zn in F_ethanol_ or F_d-H2O_ and enhanced those in F_NaCl_ or F_HAc_. For instance, in the shoots of Hanlv under treatment S1, the proportion of Zn in F_d-H2O_ decreased by 5.1% and that in F_HAc_ increased by 7.9% compared to the control. In contrast, the application of ZnNa_2_EDTA increased the proportions of Zn in F_ethanol_ or F_d-H2O_ and reduced those in F_NaCl_ or F_HAc_. For example, in the shoots of Huajun 2 under treatment E2, the proportion of Zn in F_ethanol_ increased by 8.8% and that in F_NaCl_ declined by 10.9%.

The opposing effects of ZnSO_4_ and ZnNa_2_EDTA on the chemical forms of Cd and Zn can be attributed to their different accompanying anions. For ZnSO_4_, after the sulfate anion is absorbed by the leaves of pakchoi, plants can assimilate it and synthesize S-containing amino acids (cysteine and methionine), glutathione, phytochelatins, and metallothioneins, which play key roles as metal chelators in Cd and Zn detoxification [34,35]. Therefore, the application of ZnSO_4_ might enhance the synthesis of these peptides or proteins and their chelation of Cd and Zn, resulting in increases in Cd and Zn in F_NaCl_. Consistent with this result, Lu et al. [36] observed that, with increasing ZnSO_4_ addition levels, the proportions of Cd in F_NaCl_ and F_HAc_ in the roots of wheat increased, whereas those in F_ethanol_ and F_d-H2O_ declined. Recently, a study using the XANES revealed that foliar-applied ZnEDTA could be absorbed and transported in its pristine form in wheat leaves [37]. Similarly, in the current study, ZnNa_2_EDTA might be taken up by the leaves of pakchoi; hence, the amount of the soluble form of Zn increased. Because of the equivalent affinities of Cd and Zn with EDTA [38], part of the absorbed EDTA might chelate Cd, thereby increasing the amount of the soluble Cd complex that can be extracted with deionized water. Overall, ZnSO_4_ application transformed Cd and Zn in the shoots of pakchoi from soluble chemical forms into insoluble forms, whereas ZnNa_2_EDTA application had the opposite effect.

### 3.3. Cd Bioaccessibility in Pakchoi Shoots and Health Risk Assessment of Cd

**Figure 2:**
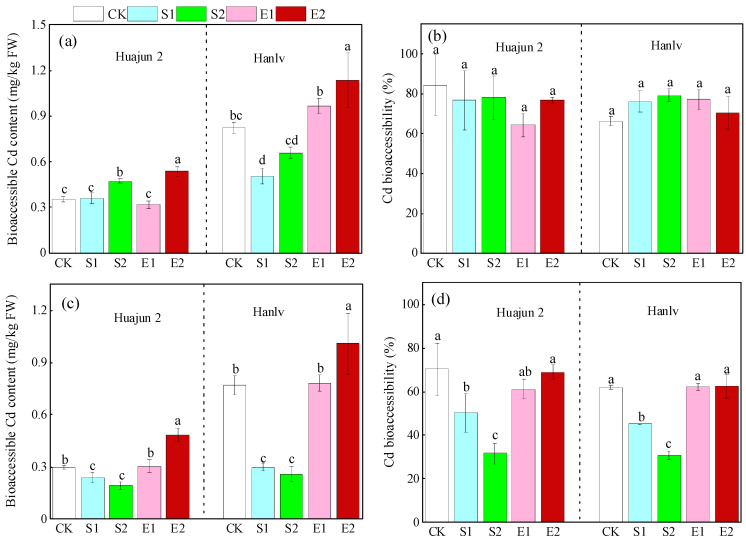
Bioaccessible content and bioaccessibility of Cd in the gastric (**a**,**b**) and small intestinal (**c**,**d**) phases in the shoots of the two cultivars of pakchoi. Different lowercase letters within the same cultivar denote significant differences amongst different Zn treatments at *p* < 0.05. CK, S1, S2, E1, and E2 represent foliar treatments applied with deionized water, 4 mM ZnSO_4_, 12 mM ZnSO_4_, 1.33 mM ZnNa_2_EDTA, and 4 mM ZnNa_2_EDTA, respectively.

Compared to the control, the application of high-dose ZnSO_4_ significantly raised the bioaccessible Cd concentration in Huajun 2 in the gastric phase by 33.0%, while the application of low- and high-dose ZnSO_4_ significantly reduced the bioaccessible Cd concentrations in Huajun 2 in the small intestinal phase by 20.3% and 35.0%, respectively (Figure 2). The application of high-dose ZnNa_2_EDTA caused significant increases in the bioaccessible Cd concentrations in Huajun 2 in the gastric and small intestinal phases (51.2% and 63.1%, respectively). For Hanlv, the application of ZnSO_4_ significantly reduced the bioaccessible Cd concentrations in the two digestion phases by 38.7–66.4%, except for the bioaccessible Cd in the gastric phase under treatment S2. Conversely, the application of high-dose ZnNa_2_EDTA significantly increased bioaccessible Cd concentrations in the gastric and small intestinal phases by 37.9% and 31.1%, respectively. In the gastric phase, Zn application had no significant effect on Cd bioaccessibility. In the small intestinal phase, the application of ZnSO_4_ significantly reduced the Cd bioaccessibility by 16.9–38.7%, whereas the application of ZnNa_2_EDTA had no significant effect. In short, ZnSO_4_ application significantly reduced Cd bioaccessibility compared to high-dose ZnNa_2_EDTA application, indicating different mechanisms of action between these Zn forms.

In this study, the Cd bioaccessibility in pakchoi ranged from 64.3% to 84.4% and 30.9% to 70.4% in the gastric and small intestinal phases, respectively. These results are consistent with those of a previous study, which showed that the average Cd bioaccessibility in pakchoi in the gastric and small intestinal phases were 68.2% and 36.8%, respectively [9]. Our previous study indicated that the application of ZnSO_4_ at 12.2 mM significantly reduced the Cd bioaccessibility in pakchoi by 24.8–59.2% [39]. A field trial reported that the application of ZnSO_4_ (12 mM) caused a significant decrease (14.1%) in Cd bioaccessibility in wheat [12]. Another field experiment showed that the application of a synthesized Zn fertilizer decreased the bioaccessible Cd concentration in a high-Cd-accumulating cultivar of water spinach by 9.2% [11]. The results of these previous studies agree well with those of the present study, which indicates that the foliar application of ZnSO_4_ could play a consistent role in reducing Cd bioaccessibility and has the potential to reduce Cd toxicity in edible crops.

**Figure 3:**
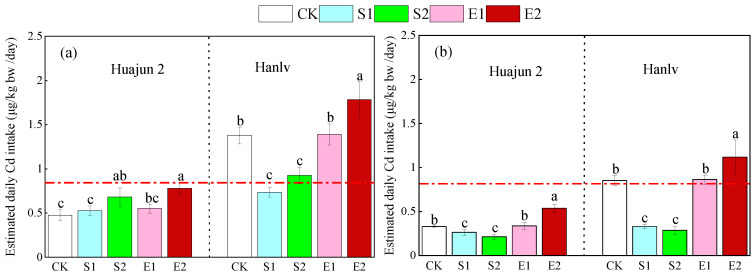
Estimated daily Cd intake based on total Cd content (**a**) and bioaccessible Cd content (**b**) from consumption of the shoots of the two cultivars of pakchoi. Different lowercase letters within the same cultivar denote significant differences amongst different Zn treatments at *p* < 0.05. The red dash lines denote the threshold value of Cd intake (0.83 μg/kg bw/day) set by JECFA. CK, S1, S2, E1, and E2 represent foliar treatments applied with deionized water, 4 mM ZnSO_4_, 12 mM ZnSO_4_, 1.33 mM ZnNa_2_EDTA, and 4 mM ZnNa_2_EDTA, respectively.

To assess the health risks of Cd to adults via pakchoi consumption, EDI values were calculated based on total Cd and bioaccessible Cd. The EDI values based on total Cd ranged from 0.48 to 1.39 μg/kg bw/day (Figure 3). For Huajun 2, the application of high-dose ZnSO_4_ and ZnNa_2_EDTA significantly raised the EDI values by 43.5% and 63.9%, respectively. Regarding Hanlv, the application of ZnSO_4_ significantly reduced the EDI values by 32.9–46.7%, while the application of high-dose ZnNa_2_EDTA significantly increased the EDI values by 29.5%. The EDI values based on bioaccessible Cd varied between 0.21 and 1.12 μg/kg bw/day. The application of ZnSO_4_ caused significant decreases in the EDI values (20.3–66.4%), while the application of high-dose ZnNa_2_EDTA significantly raised the EDI values by 31.1–63.1%. In short, the EDI values based on total Cd were markedly higher than those based on bioaccessible Cd, indicating an exaggeration of the health risk when the total Cd was considered. According to the guidelines established by the Joint Food and Agriculture Organization of the United Nations/World Health Organization Expert Committee on Food Additives (JECFA), the provisional tolerable monthly intake of Cd is 25 μg/kg bw, which is 0.83 μg/kg bw/day for the provisional tolerable daily intake (PTDI) [40]. In this study, all the EDI values of Huajun 2 were lower than the PTDI value, suggesting an acceptable health risk level. For Hanlv, the EDI values under treatment S1 were lower than the PTDI value, indicating the significant reducing effect of ZnSO_4_ on the dietary Cd intake from pakchoi.

### 3.4. Zn Bioaccessibility in Pakchoi Shoots

**Figure 4:**
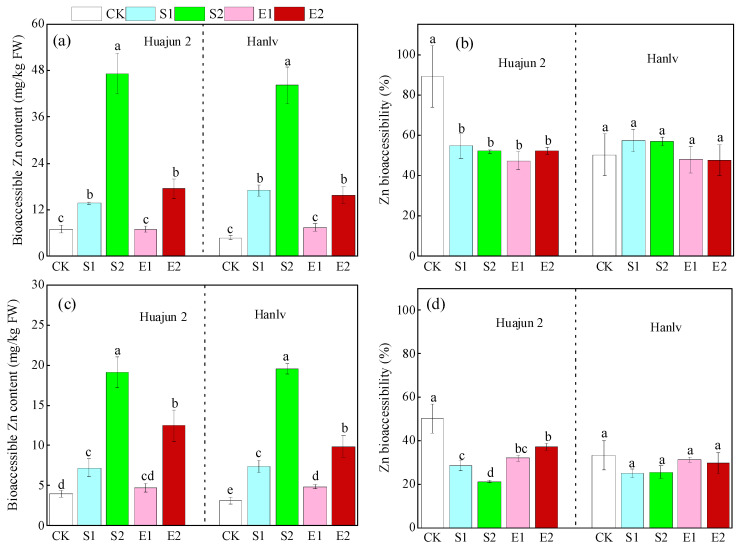
Bioaccessible content and bioaccessibility of Zn in the gastric (**a**,**b**) and small intestinal (**c**,**d**) phases in the shoots of the two cultivars of pakchoi. Different lowercase letters within the same cultivar denote significant differences amongst different Zn treatments at *p* < 0.05. CK, S1, S2, E1, and E2 represent foliar treatments applied with deionized water, 4 mM ZnSO_4_, 12 mM ZnSO_4_, 1.33 mM ZnNa_2_EDTA, and 4 mM ZnNa_2_EDTA, respectively.

The bioaccessible concentrations and bioaccessibility of Zn in pakchoi shoots under different treatments are shown in Figure 4. The application of ZnSO_4_ significantly raised the bioaccessible Zn concentrations in the gastric and small intestinal phases by 1.0–8.3- and 0.8–5.3-fold, respectively. Similarly, the application of high-dose ZnNa_2_EDTA significantly increased the bioaccessible Zn concentrations in the gastric and small intestinal phases by 1.5–2.3- and 2.0–2.2-fold, respectively. Meanwhile, foliar Zn application significantly decreased the Zn bioaccessibility in Huajun 2 in the gastric and small intestinal phases by 34.4–41.7% and 13.0–29.1%, respectively. For Hanlv, foliar Zn application had no significant influence on Zn bioaccessibility in the two digestion phases.

Our results show that the Zn bioaccessibility in pakchoi were 47.4–89.1% and 20.1–50.2% in the gastric and small intestinal phases, respectively. Similarly, Mnisi et al. [41] found that the Zn bioaccessibility in three leafy vegetables ranged from 20.0% to 60.0% and 10.0% to 50.0% in the gastric and small intestinal phases, respectively. Our previous study showed that the application of ZnSO_4_ (12.2 mM) and ZnNa_2_EDTA (3.7 mM) significantly decreased the Zn bioaccessibility in pakchoi by 9.7–50.7% and 17.5–34.0%, respectively, which is line with the results of the present work [39]. Tao et al. [12] found that the application of ZnSO_4_ (12 mM) significantly raised the Zn bioaccessibility in wheat in the gastric phase by 12.2%, and significantly reduced that in the small intestinal phase by 7.9%. Tang et al. [11] found that the application of a synthesized Zn fertilizer could significantly increase the bioaccessible concentration and bioaccessibility of Zn in water spinach. Some of the results of these two previous studies agree with the present study, while others disagree. The differences in these results indicate that the influence of foliar Zn application on Zn bioaccessibility in crops might vary depending on the crop species and the form and dose of the applied Zn.

### 3.5. Relationships between Chemical Forms and Bioaccessibility of Cd and Zn

The result of the correlation analysis (Figure S2) indicates that there were significant positive relationships between the bioaccessible Cd concentrations in the gastric and small intestinal phases and the concentrations of Cd in F_ethanol_ and F_d-H2O_ (*p* < 0.01). Significant positive relationships were also observed between bioaccessible Zn concentrations in the two digestion phases and the concentrations of Zn in the five chemical forms (*p* < 0.01). Cd bioaccessibility in the small intestinal phase was significantly positively correlated with the proportion of Cd in F_d-H2O_ and negatively correlated with that in F_NaCl_ (*p* < 0.01). Zn bioaccessibility in the small intestinal phase was significantly positively correlated with the proportion of Zn in F_ethanol_ (*p* < 0.01) and negatively correlated with that in F_NaCl_ (*p* < 0.05).

A multiple linear regression analysis with a stepwise procedure was conducted to identify the critical factors influencing the bioaccessibility of Cd and Zn (Table S2). The results show that the bioaccessible Cd concentrations in the gastric and small intestinal phases were significantly influenced by the concentrations of Cd in F_d-H2O_ and F_NaCl_. Meanwhile, the bioaccessible Zn concentration was significantly affected by the concentrations of Zn in F_NaCl_ and F_HCl_ in the gastric phase and by those in F_ethanol_ and F_NaCl_ in the small intestinal phase. 

RDA was performed to compare the effects of different chemical forms of Cd and Zn on their bioaccessibility. The first canonical axis (RDA1) and the second canonical axis (RDA2) accounted for 90.51% and 3.43% of the total variance of the bioaccessible Cd concentration, respectively (Figure S3). The concentrations of Cd in F_d-H2O_ and F_NaCl_ had the most important effects on the bioaccessible Cd concentration, which explained 78.1% and 14.9% of the variability, respectively. The variability in the bioaccessible Zn concentration was explained mainly by RDA1 (95.89%, Figure S4). The concentrations of Zn in F_NaCl_ and F_ethanol_ had the largest contributions to the bioaccessible Zn concentration (90.5% and 6.0%, respectively).

**Figure 5:**
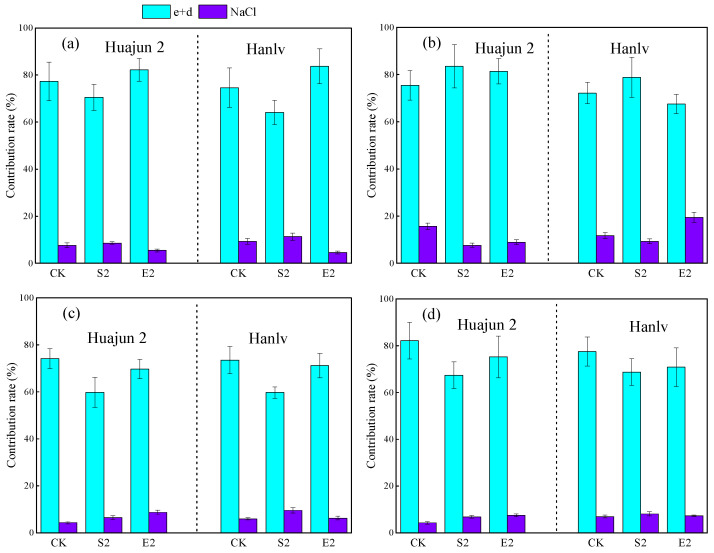
The contribution rates of F_ethanol_ and F_d-H2O_ (e+d), as well as F_NaCl_ (NaCl) to bioaccessible Cd (**a**,**b**) and Zn (**c**,**d**) in the gastric (**a**,**c**) and small intestinal (**b**,**d**) phases in the shoots of the two cultivars of pakchoi. CK, S2, and E2 represent foliar treatments applied with deionized water, 12 mM ZnSO_4_, and 4 mM ZnNa_2_EDTA, respectively.

To sum up, the results of the above statistical analyses indicate that Cd and Zn in F_ethanol_, F_d-H2O_, and F_NaCl_ were the main contributors to bioaccessible Cd and Zn in pakchoi. To confirm this statement, the contributions of the three chemical forms to bioaccessible Cd and Zn were further assessed based on the decreases in the bioaccessible concentrations of Cd and Zn after removing the related fractions (Figure 5). The CR of F_ethanol_ and F_d-H2O_ to bioaccessible Cd ranged from 64.1% to 83.7%, averaging 75.4% in the gastric phase, and from 67.5% to 83.5%, averaging 76.5% in the small intestinal phase. The CR of F_ethanol_ and F_d-H2O_ to bioaccessible Zn were 59.7–74.2%, with a mean of 68.0%, and 67.4–82.1%, with a mean of 73.6%, in the gastric and small intestinal phases, respectively. In comparison, the CR of F_NaCl_ to bioaccessible Cd were 4.6–11.3%, with a mean of 7.8%, and 7.7–19.5%, with a mean of 12.1%, in the gastric and small intestinal phases, respectively. The CR of F_NaCl_ to bioaccessible Zn were 4.3–9.6%, with a mean of 6.9%, and 4.3–8.1%, with a mean of 6.8%, in the gastric and small intestinal phases, respectively. Overall, in the shoots of the tested pakchoi cultivars, Cd and Zn in F_ethanol_ and F_d-H2O_ contributed to more than 59% of the bioaccessible Cd and Zn, while Cd and Zn in F_NaCl_ accounted for <19.5%, which indicates that F_ethanol_ and F_d-H2O_ were the primary sources of bioaccessible Cd and Zn. Since F_ethanol_ and F_d-H2O_ are soluble fractions, in theory, Cd and Zn in these forms could be more easily released than those in other forms during the gastrointestinal digestive process [42].

Owing to the principal contributions of F_ethanol_ and F_d-H2O_ to bioaccessible Cd and Zn in pakchoi shoots, the changes in the two chemical forms as influenced by foliar Zn application could lead to variations in the bioaccessibility of Cd and Zn. For Cd, the application of ZnSO_4_ tended to reduce the concentrations of Cd in F_ethanol_ and F_d-H2O_ by 6.4–19.9% and 4.3–71.1%, respectively, whereas the application of high-dose ZnNa_2_EDTA significantly raised the concentrations of Cd in F_ethanol_ and F_d-H2O_ by 0.5–1.9- and 0.9–3.5-fold, respectively. Correspondingly, the bioaccessible Cd concentrations in the small intestinal phase under treatments S1 and S2 decreased significantly by 20.3–66.4%, while the bioaccessible Cd concentrations under treatment E2 increased significantly by 31.1–63.1%. For Zn, the application of ZnSO_4_ and high-dose ZnNa_2_EDTA significantly increased the concentrations of Zn in F_ethanol_ and F_d-H2O_ by 1.0–6.4-fold. Correspondingly, the bioaccessible Zn concentrations under treatments S1, S2, and E2 increased significantly by 0.8–8.3-fold. These results suggest that foliar Zn application can affect Cd and Zn bioaccessibility in pakchoi mainly by modulating the concentrations of Cd and Zn in F_ethanol_ and F_d-H2O_.

## 4. Conclusions

This work suggests that ZnSO_4_ is an efficient foliar fertilizer because it could significantly reduce the accumulation, bioaccessibility, and attendant health risks of Cd and significantly increase the total and bioaccessible concentrations of Zn in pakchoi. This result provides scientific support for the development of more efficient measures to produce safe and high-quality leafy vegetables from Cd-polluted soils. The significant positive correlations between bioaccessible Cd and Zn and F_ethanol_ and F_d-H2O_, as well as the main contributions of F_ethanol_ and F_d-H2O_ to Cd and Zn bioaccessibility suggest that changes in the two chemical forms, as influenced by foliar Zn application, could lead to changes in the bioaccessible Cd and Zn. This finding enhances our understanding of the mechanisms by which foliar Zn application influences Cd and Zn bioaccessibility. One limitation of this study is the short duration of the pot experiment, which may not capture the long-term effect of Zn application on metal bioaccessibility. Future studies should explore the long-term impact of foliar Zn application on Cd and Zn bioaccessibility under field conditions and across different crop species.

## Figures and Tables

**Table 1:** Concentrations of Cd in different chemical forms in the shoots of two cultivars of pakchoi.

Chemical Forms	Treatments	Cd Concentrations (mg/kg FW)	
		Huajun 2	Hanlv
F_ethanol_	CK	0.0338 ± 0.0047 ab	0.0557 ± 0.0098 c
	S1	0.0271 ± 0.0039 b	0.0681 ± 0.0111 bc
	S2	0.0317 ± 0.0060 b	0.0501 ± 0.0100 c
	E1	0.0269 ± 0.0024 b	0.0858 ± 0.0154 b
	E2	0.0427 ± 0.0051 a	0.159 ± 0.015 a
F_d-H2O_	CK	0.107 ± 0.025 c	0.397 ± 0.072 c
	S1	0.063 ± 0.010 c	0.115 ± 0.015 d
	S2	0.103 ± 0.011 c	0.145 ± 0.021 d
	E1	0.336 ± 0.055 b	0.772 ± 0.091 b
	E2	0.485 ± 0.099 a	1.20 ± 0.17 a
F_NaCl_	CK	0.250 ± 0.030 b	0.732 ± 0.045 a
	S1	0.328 ± 0.054 ab	0.425 ± 0.030 c
	S2	0.433 ± 0.083 a	0.589 ± 0.029 b
	E1	0.114 ± 0.018 c	0.335 ± 0.026 cd
	E2	0.108 ± 0.023 c	0.235 ± 0.043 d
F_HAc_	CK	0.0327 ± 0.0031 ab	0.0491 ± 0.0065 a
	S1	0.0505 ± 0.0143 ab	0.0444 ± 0.0058 a
	S2	0.0422 ± 0.0006 ab	0.0420 ± 0.0092 a
	E1	0.0179 ± 0.0045 b	0.0468 ± 0.0132 a
	E2	0.0531 ± 0.0120 a	0.0137 ± 0.0038 a
F_HCl_	CK	0.00529 ± 0.00026 ab	0.00929 ± 0.00188 ab
	S1	0.00775 ± 0.00187 ab	0.0101 ± 0.0021 ab
	S2	0.00582 ± 0.00124 ab	0.00834 ± 0.00200 ab
	E1	0.00309 ± 0.00076 b	0.0162 ± 0.0045 a
	E2	0.0142 ± 0.0038 a	0.00364 ± 0.00047 b

F_ethanol_, F_d-H2O_, F_NaCl_, F_HAc_, and F_HCl_ represent chemical forms of Cd extracted with 80% ethanol, deionized water, 1 M NaCl, 2% acetic acid, and 0.6 M HCl, respectively. CK, S1, S2, E1, and E2 represent foliar treatments applied with deionized water, 4 mM ZnSO_4_, 12 mM ZnSO_4_, 1.33 mM ZnNa_2_EDTA, and 4 mM ZnNa_2_EDTA, respectively. The data are expressed as the mean ± standard error (*n* = 3). Different lowercase letters within the same cultivar denote significant differences amongst different Zn treatments at *p* < 0.05.

**Table 2:** Concentrations of Zn in different chemical forms in the shoots of two cultivars of pakchoi.

Chemical Forms	Treatments	Zn Concentrations (mg/kg FW)	
		Huajun 2	Hanlv
F_ethanol_	CK	2.34 ± 0.28 e	2.85 ± 0.33 e
	S1	9.03 ± 1.19 c	8.00 ± 0.54 c
	S2	15.3 ± 1.4 a	13.8 ± 0.1 a
	E1	4.36 ± 0.66 d	5.18 ± 0.44 d
	E2	12.8 ± 1.2 b	10.4 ± 0.7 b
F_d-H2O_	CK	1.59 ± 0.11 d	1.79 ± 0.23 c
	S1	3.11 ± 0.53 c	4.17 ± 0.77 b
	S2	11.8 ± 1.1 a	13.3 ± 1.3 a
	E1	3.25 ± 0.41 c	3.24 ± 0.53 bc
	E2	6.65 ± 1.04 b	11.6 ± 1.5 a
F_NaCl_	CK	2.12 ± 0.41 b	2.79 ± 0.45 b
	S1	3.56 ± 0.78 b	7.51 ± 1.28 b
	S2	35.7 ± 3.5 a	32.1 ± 6.7 a
	E1	4.93 ± 0.94 b	3.83 ± 0.76 b
	E2	5.44 ± 1.02 b	7.08 ± 1.86 b
F_HAc_	CK	1.18 ± 0.09 c	1.52 ± 0.27 c
	S1	7.15 ± 0.53 b	7.17 ± 1.42 b
	S2	22.5 ± 4.0 a	13.7 ± 0.4 a
	E1	1.38 ± 0.19 c	1.90 ± 0.17 c
	E2	5.78 ± 1.47 b	2.66 ± 0.56 c
F_HCl_	CK	0.68 ± 0.10 c	0.93 ± 0.18 c
	S1	2.47 ± 0.33 b	3.02 ± 0.39 b
	S2	5.34 ± 0.67 a	4.92 ± 0.74 a
	E1	0.86 ± 0.20 c	1.52 ± 0.37 c
	E2	2.77 ± 0.28 b	1.53 ± 0.35 c

CK, S1, S2, E1, and E2 represent foliar treatments applied with deionized water, 4 mM ZnSO_4_, 12 mM ZnSO_4_, 1.33 mM ZnNa_2_EDTA, and 4 mM ZnNa_2_EDTA, respectively. The data are expressed as the mean ± standard error (*n* = 3). Different lowercase letters within the same cultivar denote significant differences amongst different Zn treatments at *p* < 0.05.

## Data Availability

The original contributions presented in the study are included in the article/Supplementary Materials, further inquiries can be directed to the corresponding author.

## Supplementary Materials

The following supporting information can be downloaded at: https://www.mdpi.com/article/10.3390/foods13152430/s1. Table S1: Basic physicochemical characteristics of the experimental soil. Table S2: The relationships between bioaccessible content of Cd and Zn in the gastric and small intestinal phases and Cd and Zn content in different chemical forms as given by stepwise multiple linear regression analysis. Figure S1: Proportions of Cd and Zn in different chemical forms in the shoots of the two cultivars of pakchoi. Figure S2: Correlation coefficients for the relationships between bioaccessible content of Cd and Zn in the gastric and small intestinal phases and Cd and Zn content in different chemical forms, and for the relationships between Cd and Zn bioaccessibility in the gastric and small intestinal phases and Cd and Zn proportions in different chemical forms. Figure S3: Redundancy analysis of the correlations between bioaccessible Cd content in the gastric and small intestinal phases and Cd content in different chemical forms. Figure S4: Redundancy analysis of the correlations between bioaccessible Zn content in the gastric and small intestinal phases and Zn content in different chemical forms.
