# Supplementary material for: Influence of Foliar Zinc Application on Cadmium and Zinc Bioaccessibility in Brassica chinensis L.: In Vitro Digestion and Chemical Sequential Extraction
Source: Foods. 2024 Aug 1;13(15):2430. doi: 10.3390/foods13152430 (PMC11311326; doi:10.3390/foods13152430)
Supplement: Supplementary file 1 [file foods-13-02430-s001.zip › foods-3096466-supplementary.pdf]

Supporting materials

Table S1. Basic Physicochemical Characteristics of the Experimental Soil

pH	CEC (cmol/kg)	OM (g/kg)	Ah N (mg/kg)	Available P (mg/kg)	Available K (mg/kg)	Total Cd (mg/kg)	Total Zn (mg/kg)
7.45	15.2	6.57	80.5	21.4	97.5	2.56	58.6

CEC: cation exchange capacity; OM: organic matter; Ah N: alkali-hydrolysable N.

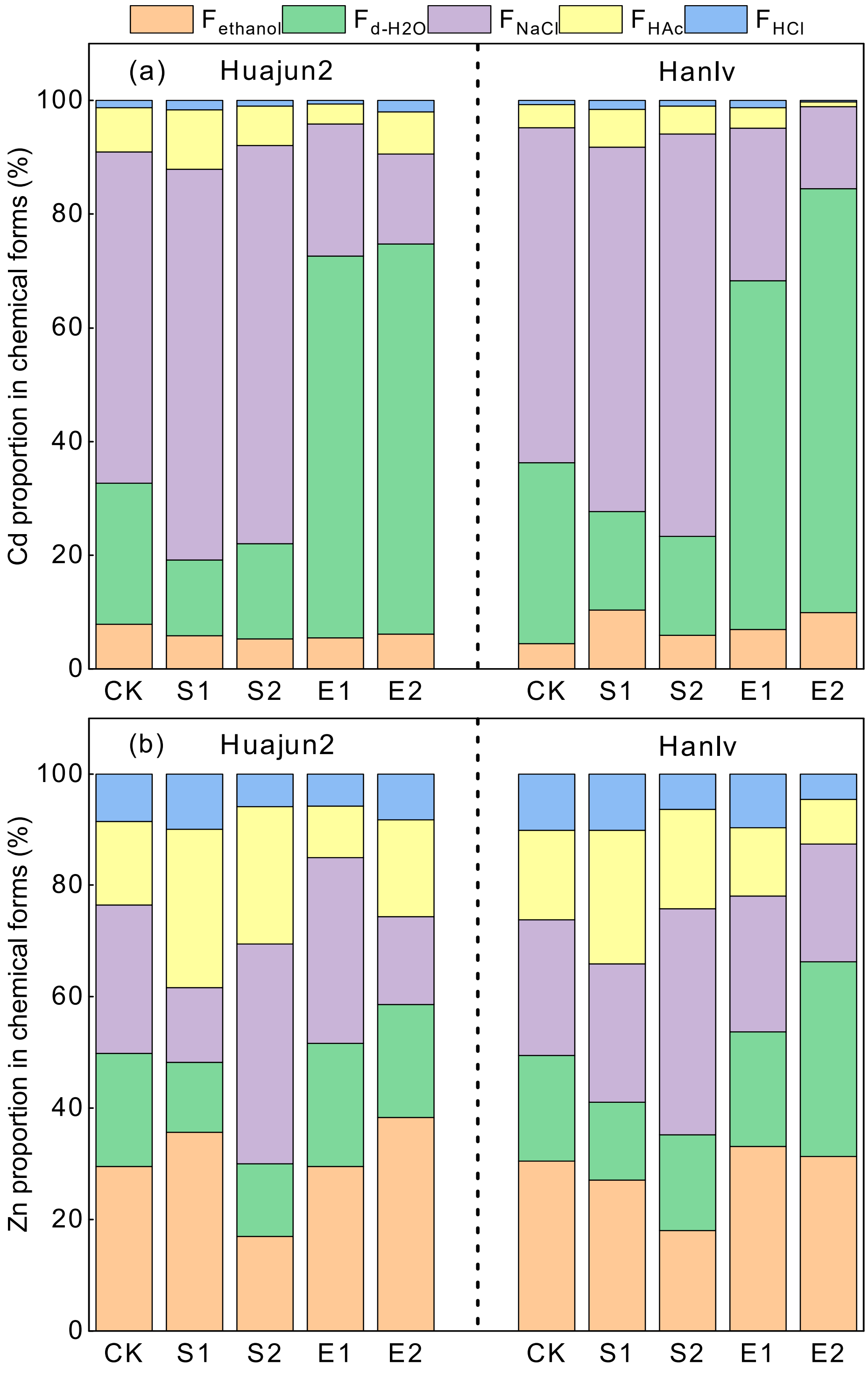

Figure S1. Proportions of Cd (a) and Zn (b) in different chemical forms in the shoots of the two cultivars of pakchoi. $F_{ethanol}$, $F_{d\text{-}H2O}$, F_{NaCl}, F_{HAc}, and F_{HCl} represent chemical forms of Cd or Zn extracted with 80% ethanol, deionized water, 1 M NaCl, 2% acetic acid, and 0.6 M HCl, respectively. CK, S1, S2, E1, and E2 represent foliar treatments

applied with deionized water, 4 mM $ZnSO_4$, 12 mM $ZnSO_4$, 1.33 mM $ZnNa_2EDTA$, and 4 mM $ZnNa_2EDTA$, respectively.

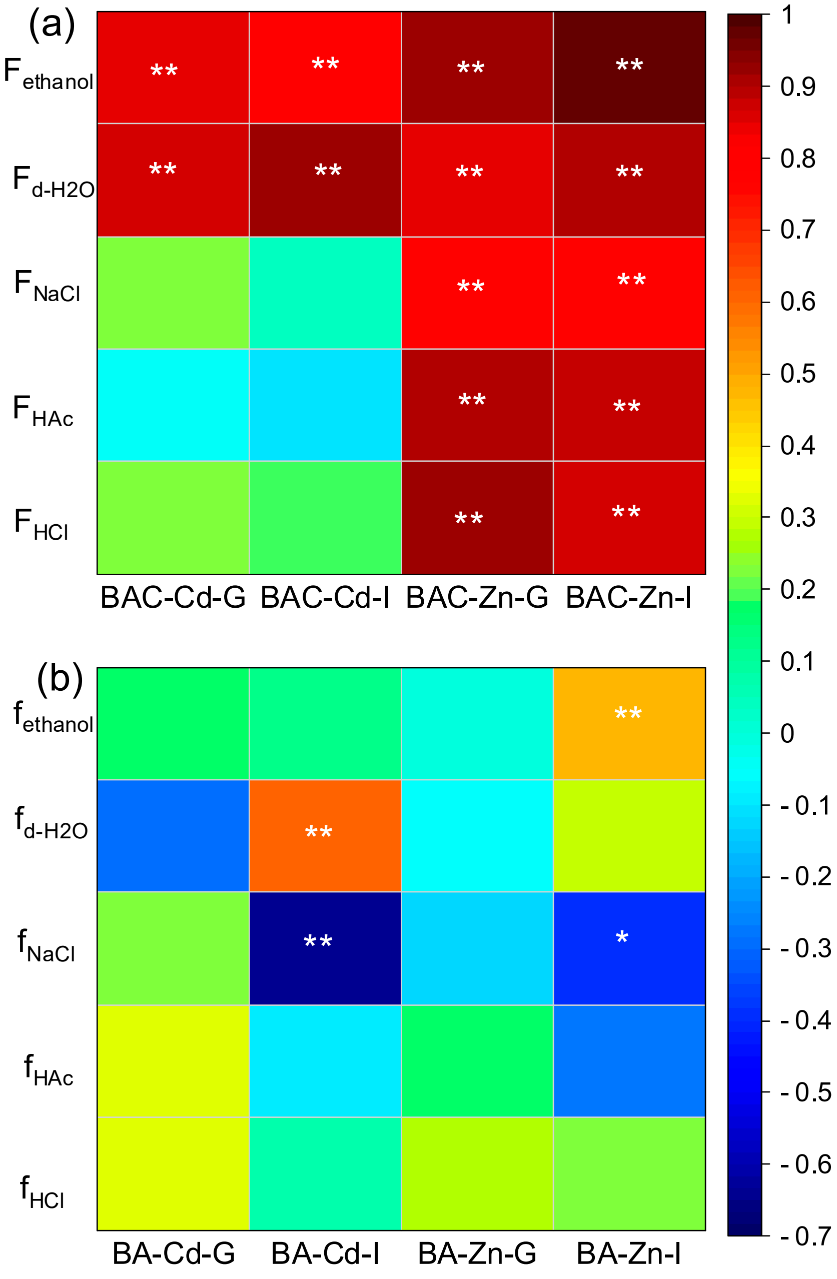

Figure S2. Correlation coefficients for the relationships between bioaccessible content (BAC) of Cd and Zn in the gastric (G) and small intestinal (I) phases and Cd and Zn content in different chemical forms (a), and for the relationships between Cd and Zn

bioaccessibility (BA) in the gastric and small intestinal phases and Cd and Zn proportions in different chemical forms (b). $f_{ethanol}$, $f_{d\text{-}H2O}$, f_{NaCl}, f_{HAc}, and f_{HCl} represent the proportions of Cd or Zn extracted with 80% ethanol, deionized water, 1 M NaCl, 2% acetic acid, and 0.6 M HCl, respectively. ** and * represent 1% and 5% levels of significance (two-tailed), respectively.

Table S2. The relationships between bioaccessible content of Cd (BAC-Cd) and Zn (BAC-Zn) in the gastric and small intestinal phases and Cd and Zn content in different chemical forms as given by stepwise multiple linear regression analysis (*n* = 30).

The gastric phase	The small intestinal phase
BAC-Cd = 0.098+ 0.745 $F_{d\text{-}H2O}$ + 0.673 F_{NaCl}	BAC-Cd = 0.023+ 0.779 $F_{d\text{-}H2O}$ + 0.424 F_{NaCl}
(R^2 = 0.934, $p < 0.001$)	(R^2 = 0.916, $p < 0.001$)
BAC-Zn = 1.090+ 0.659 F_{NaCl} + 4.222 F_{HCl}	BAC-Zn= 0.524+ 0.766 $F_{ethnaol}$+ 0.215 F_{NaCl}
(R^2 = 0.968, $p < 0.001$)	(R^2 = 0.950, $p < 0.001$)

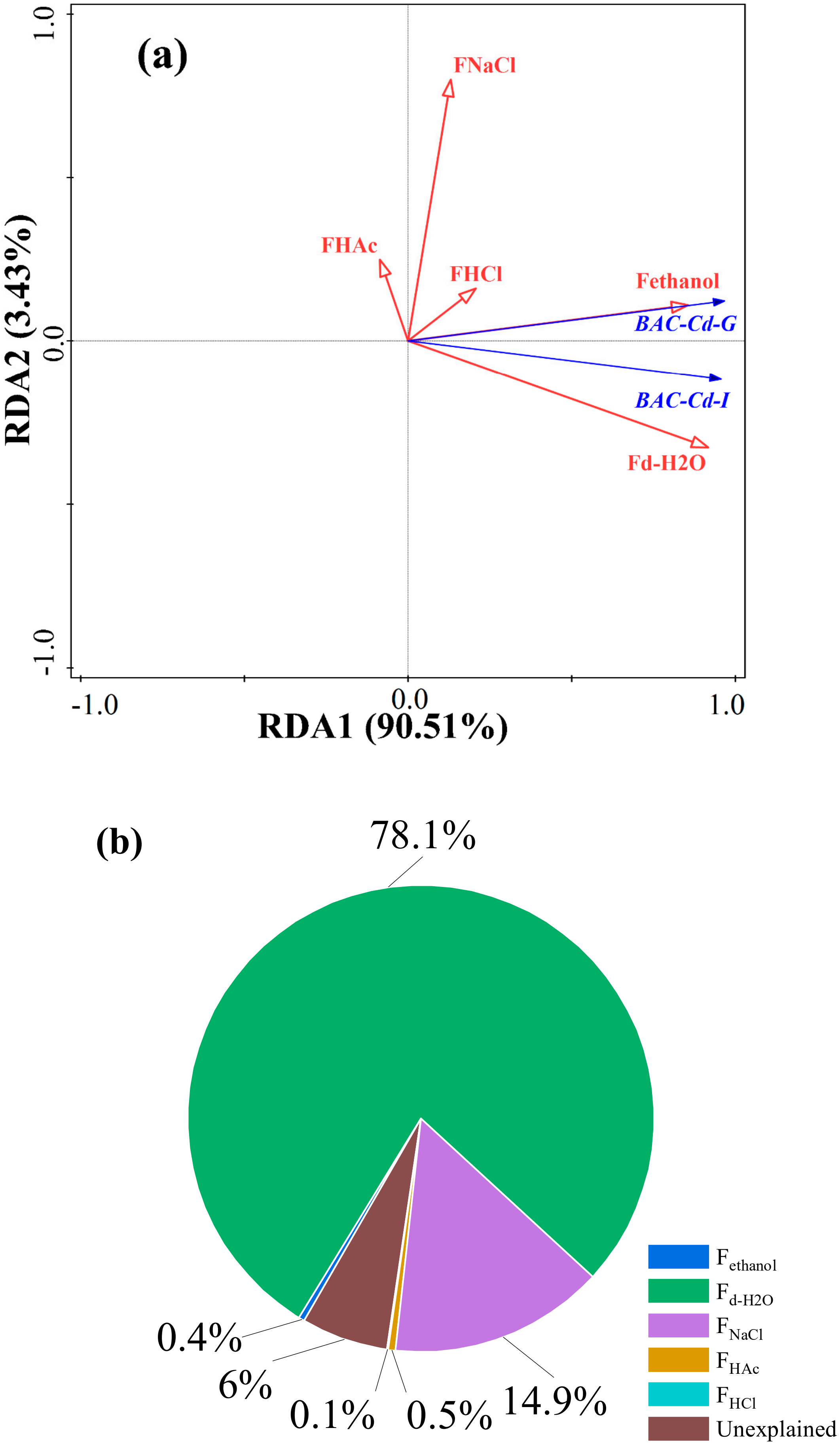

(a)
FNaCl
FHAc
FHCl
Fethanol
BAC-Cd-G
BAC-Cd-I
Fd-H2O
RDA2 (3.43%)
RDA1 (90.51%)
1.0
0.0
-1.0
-1.0
0.0
1.0
(b)
78.1%
0.4%
6%
0.1%
0.5%
14.9%
F ethanol
F d-H2O
F NaCl
F HAc
F HCl
Unexplained

Figure S3. Redundancy analysis of the correlations between bioaccessible Cd content (BAC-Cd) in the gastric (G) and small intestinal (I) phases and Cd content in different chemical forms. The length of arrows representing chemical forms and the cosine of the angle between the arrows of bioaccessible Cd content and the arrows of chemical forms indicate the correlational strength between bioaccessible Cd content and chemical forms (a). The explained variations were obtained from the partial redundancy analysis investigating the effects of Cd chemical forms on bioaccessible Cd content (b).

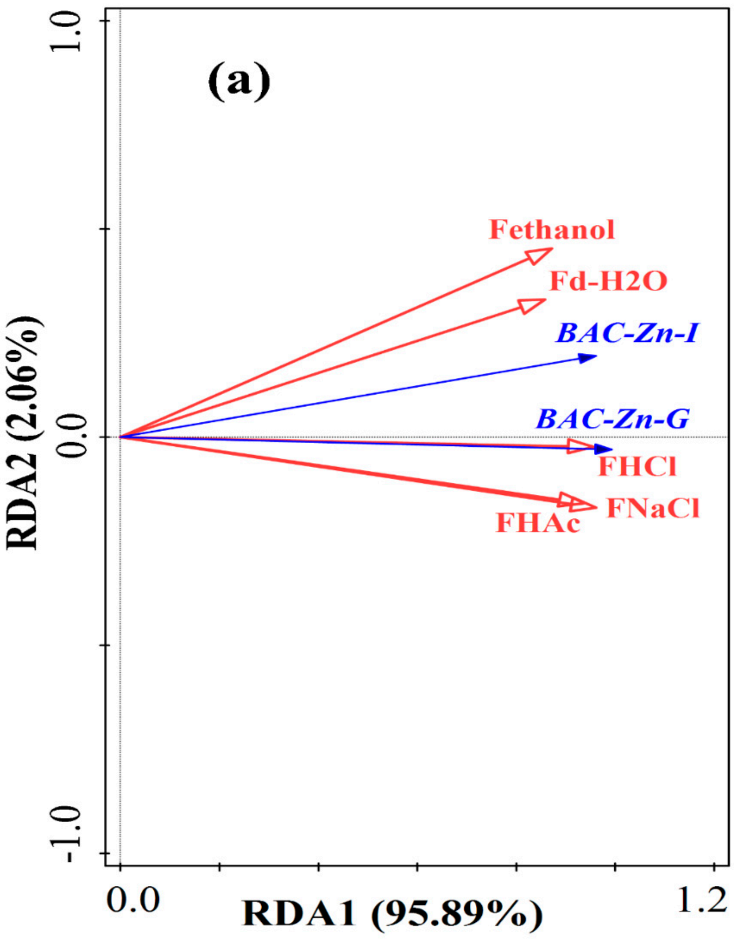

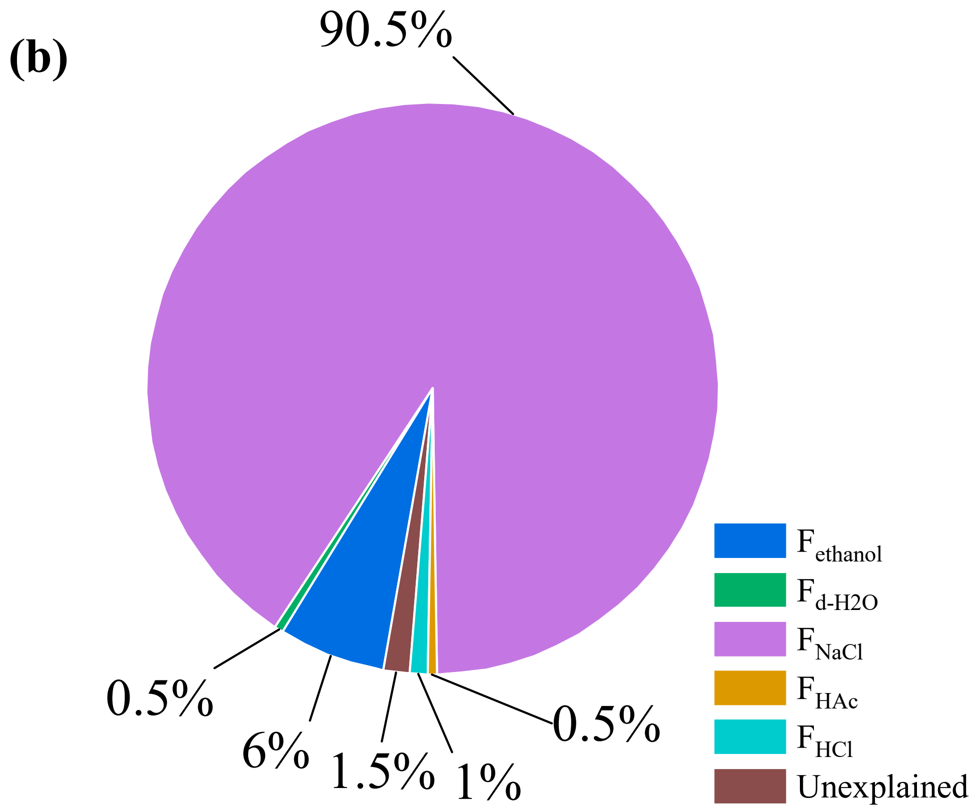

Figure S4. Redundancy analysis of the correlations between bioaccessible Zn content (BAC-Zn) in the gastric (G) and small intestinal (I) phases and Zn content in different chemical forms. The length of arrows representing chemical forms and the cosine of the angle between the arrows of bioaccessible Zn content and the arrows of chemical forms indicate the correlational strength between bioaccessible Zn content and chemical forms (a). The explained variations were obtained from the partial redundancy analysis investigating the effects of Zn chemical forms on bioaccessible Zn content (b).
